# Patterns and Predictors of Non-Prescription Medicine Use among Malaysian Pharmacy Patrons: A National Cross Sectional Study

**DOI:** 10.1371/journal.pone.0059231

**Published:** 2013-04-03

**Authors:** Mohamed Azmi Hassali, Abdul Haniff Mohamad Yahaya, Asrul Akmal Shafie, Fahad Saleem, Gin Nie Chua, Hisham Aljadhey

**Affiliations:** 1 Discipline of Social and Administrative Pharmacy, School of Pharmaceutical Sciences, Universiti Sains Malaysia, Pinang, Malaysia; 2 Department of Pharmacy, University of Balochistan, Quetta, Pakistan; 3 College of Pharmacy, King Saud University, Riyadh, Saudi Arabia; UCL Institute of Child Health, University College London, United Kingdom

## Abstract

**Objective:**

The study aims to evaluate the predictors of non-prescription medicine purchasing patterns among pharmacy patrons in Malaysia.

**Methods:**

A cross-sectional nationwide study was undertaken in 2011 in sixty randomly selected community pharmacies across 14 Malaysian states. A pharmacy exit survey was conducted over a 6-month period across Malaysia. A one-stage random cluster sampling technique was employed as there was no national sampling framework available for conducting this survey. Face-to-face interviews using a validated and pre-tested questionnaire were conducted by trained data collectors. The non-prescription medicine purchasing pattern was explored and analysed descriptively. Chi-square/Fisher exact test was used to determine the association between study variables. Multinomial logistic regression analysis was used to determine the predictors of type of non-prescription medicine purchased.

**Results:**

A total of 2729 pharmacy patrons agreed to participate in 60 selected pharmacy outlets. A total of 3462 non-prescription medicine were purchased during the study period with an average of 1.3 medicines per participant. Most of the non-prescription medicine purchased was meant for alimentary tract and metabolism (31.8%), followed by respiratory system (19.4%) and musculoskeletal system (15.8%) usage. Factors found to be associated with the choice of non-prescription medicine purchased were age group [χ2 = 170.75, (df = 57), p<0.01], locality [χ2 = 48.16, (df = 19), p<0.01], gender [χ2 = 32.93, (df = 13), p = 0.002], ethnic group [χ2 = 118.89, (df = 39), p<0.01] and type of occupation [χ2 = 222.434, (df = 117), p<0.01]. Non-prescription medicine purchased defined about 20% of the variance in the combination of predictors such as locality, gender, age, ethnicity, type of occupation and household income.

**Conclusion:**

The predictors for selection of non-prescription medicine were locality (urban or rural), gender, age, ethnicity, type of occupation and household income per month. Future studies need to explore the safety and effectiveness of using these non-prescription medicines.

## Introduction

In recent years, there has been an increasing trend for self-medication with non-prescription medicines (NPM). In parallel, more products have been deregulated for purchase without a prescription [1]. Population-based survey in developed countries such as Australia, Scotland, United Kingdom and other Asian countries like Taiwan and Singapore found that between a half and two-thirds of the population used NPM, including complementary and over-the-counter medicines (OTC) [2]–[8].

A study in the United States found that the average profit margin from NPM sales ranged from 32–36% compared to only 25% profit from the sales of prescription medicines [9]. In Malaysia, the lack of dispensing rights might encourage community pharmacists to focus on sales of NPM. Indeed, there was increase in Malaysian total healthcare expenditure on OTC medicine from Ringgit Malaysia (RM) 1.1 billion in 2001 to RM 1.5 billion in 2006 [10]. Unfortunately, the report focused more on sales of the product than the population’s utilization of NPM.

Knowledge of NPM utilization is required to determine the self-medication behaviour of society. The growth of OTC healthcare in Malaysia is believed to have been driven by greater willingness to self-medicate. The increased public interest in self-medication might be due to general frustrations with conventional therapy, changes in purchasing power, the dramatic increase in the number of products available, and the marked reduction in the threshold of tolerance to symptoms. Gaining an understanding of the types of NPM utilized by the general public would provide baseline information on the self-medication status of the population.

In Malaysia, NPM encompasses pharmacist only medicines (POM), OTC medicines and traditional medicines. Pharmacist only medicines comprise of group C poisons under the Poison Schedule of Poison Act 1952, whereby they can only be sold by registered pharmacists in Malaysia [11]. Only community pharmacy outlets employing a registered pharmacist are eligible to sell such medicines. Over-the-counter and traditional medicines are sold by any retailer in Malaysia. Thus, the availability of NPM in community pharmacies and other retail outlets depends on their legal status. Within this context, NPM utilization by Malaysians should be evaluated in the community pharmacy setting to have a better insight of the purchasing pattern. Thus, the aim of this study is to evaluate the predictors of non-prescription medicine purchasing patterns among pharmacy patrons in Malaysia.

## Methodology

### Design

The study was designed as a nationwide, questionnaire based, cross-sectional survey targeting community pharmacy patrons across Malaysia. A pharmacy “exit survey” was developed and administered to consenting community pharmacy patrons of 60 randomly selected community pharmacies across 14 Malaysian states.

### Sampling and Sample Size

The projected sample size of respondents needed was 2564 pharmacy patrons based on a 95% confidence interval and margin of error of 5% with an estimated 50% response rate [12]. The study used the simplified cluster sampling method, which allowed the random selection of 60 community pharmacy patrons in 10 clusters from 14 selected Malaysian states. This method is suitable for use by teams of data collectors who lack skills in sampling, and where comprehensive sampling frameworks are not available. The method does not randomly select the consumer, but instead requires the interviewer to follow a particular path through the community, sampling all community pharmacy consumers for 10 days. Hence, the selection of participants was based on convenience rather than being random. Although the method suffers some bias, its validity is good, and it has been widely used by teams of researchers to collect data for a wide range of health and social issues [13].

### Data Collection Method

Undergraduate students from School of Pharmaceutical Sciences, Universiti Sains Malaysia were provided half a day training regarding data collection procedures. The training was conducted by the research team. The data collectors interviewed the community pharmacy patrons who agreed to participate upon exiting the pharmacy outlets. The study was conducted from March 2009 to September 2009. The questionnaires consisted of two parts: demographic data and information on NPM purchased. The names of NPM purchased were collected and categorized according to the anatomical therapeutic chemical (ATC) classification up to level two (main therapeutic group). The ATC system is used for the classification of drugs and is controlled by the World Health Organization (WHO). This pharmaceutical coding system divides drugs into different groups according to the organ or system on which they act and/or their therapeutic and chemical characteristics. Each bottom-level ATC code stands for a pharmaceutically used substance, or a combination of substances, in a single indication.

The data collection tool was validated by a group of reviewers consisting of two senior lecturers stationed as School of Pharmaceutical Sciences, Universiti Sains Malaysia, two community pharmacists working in Penang Island and two pharmacists from Malaysian Ministry of Health. In addition, the data collection tool was also declared reliable as the alpha value was in acceptable ranges (0.75).

### Data Analysis

In the descriptive analysis, the continuous variables were represented as means and standard deviation while the categorical variables were represented as frequency and percentages. Chi-square/Fischer Exact analysis was used to identify the demographic factors associated with the type of NPM purchased. Multinomial logistic regression analysis was used to determine the predictors of type of non-prescription medicine purchased. The analysis was conducted by Predictive Analytics Software (PASW) v. 18. A p-value of less than 0.05 was considered as statistically significant.

### Ethical Approval

Ethical approval for the conduct of this study was given by the Malaysian Institute of Public Health (NMRR-10-1028-7676). Written consent was taken from the pharmacy owners as well as community pharmacy patrons prior to data collection. In addition, the patrons were also informed about the research initiatives, confidentiality of their responses and their right to withdraw from the study.

## Results

### Sample Characteristics

A total of 3000 questionnaires were distributed to pharmacy patrons at selected pharmacy outlets. However, only 2800 questionnaires were returned, of which 2729 were utilized for the final analysis.

The mean age of the participants was 40.25 years (SD = 13.85). The proportion of male and female participants was similar. The percentage of urban participants was higher than that of rural participants. The demographic characteristics of the participants are summarized in Table 1.

**Table 1:** Demographic profile of community pharmacy consumers (n = 2729).

Characteristics	n	Percentage (%)
***Age***		
16 to 25 years old	441	16.2
26 to 40 years old	1053	38.6
41 to 60 years old	1091	40.0
More than 60 years old	144	5.3
***Locality***		
Rural	595	21.8
Urban	2134	78.2
***Gender***		
Male	1369	50.2
Female	1360	49.8
**Ethnic**		
Malay	1435	52.6
Chinese	788	28.9
Indian	260	9.5
Other	245	9.0
**Education status**		
No formal education	113	4.1
Primary education	387	14.2
Secondary education	1427	52.3
Tertiary education	802	29.4
**Occupation**		
Senior officials & manager	161	6.0
Professionals	428	15.9
Technician & associate professional	146	5.4
Clerical works	198	7.3
Service workers, shop & market sales workers	433	16.1
Craft & related trades workers	89	3.3
Skilled agricultural & fishery workers	118	4.4
Plant & machine-operator & assemblers	72	2.7
Elementary occupation	203	7.5
Students	214	7.9
Retiree	153	5.7
Not working	479	17.8
**Number of family member**		
Mean ± standard deviation	4.99±2.21	
Median ± Inter quartile range	5.00±2.00	
**Household income per month (n = 2708)**		
Less than RM500	153	5.6
RM500 to RM1000	414	15.3
RM1001 to RM2000	647	23.9
RM2001 to RM3000	609	22.5
RM3001 to RM4000	381	14.1
RM4001 to RM5000	181	6.7
More than RM5000	323	11.9

### Types of Non-prescription Medicine Purchased

A total of 3462 NPM were purchased during the study period with mean being 1.27 (SD = 0.60) per participant. The types of NPM purchased are summarized in Table 2.

**Table 2:** Types of non-prescription medicine purchased.

Types of non-prescription medicine (ATC classification)	Frequency	Percentage
***Alimentary Tract & Metabolism***		
Stomatological Preparation	117	3.38
Drugs For Acid Related Disorders	156	4.51
Drugs For Functional Gastrointestinal Disorders	75	2.17
Bile And Liver Therapy	17	0.49
Laxatives	60	1.73
Anti diarrheal, Intestinal Anti inflammatory	65	1.88
Anti obesity Preparations, Excluding Dietary Products	29	0.84
Digestive (including Enzymes)	17	0.49
Anti Diabetics	38	1.10
Vitamins	281	8.12
Mineral Supplements	140	4.04
Appetite Stimulants	1	0.03
Other Alimentary Tract And Metabolism Products	105	3.03
***Blood & Blood Forming Organs***		
Antithrombotic Agents	14	0.40
Anti Anaemic Preparations	8	0.23
Blood Substitutes And Perfusion Solutions	6	0.17
***Cardiovascular System***		
Anti hypertensives	1	0.03
Diuretics	5	0.14
Peripheral Vasodilators	3	0.09
Vasoprotectives	3	0.09
Beta Blocking Agents	11	0.32
Calcium Channel Blockers	5	0.14
Agent Acting On The Renin-Angiotensin Systems	6	0.17
Serum Lipid Reducing Agents	80	2.31
***Dermatologicals***		
Anti fungal For Dermatological Use	93	2.69
Emollients And Protective	46	1.33
Preparations For Treatment Of Wounds And Ulcers	7	0.20
Anti pruritic (including Antihistamines, Anesthethic)	18	0.52
Anti psoriatic	6	0.17
Antibiotic And Chemotherapeutics For Dermatological Use	14	0.40
Corticosteroid, Dermatological Preparation	81	2.34
Antiseptic And Anti disinfectants	31	0.90
Medicated Dressings	4	0.12
Anti Acne Preparations	20	0.58
Other Dermatological Preparations	25	0.72
***Genito-Urinary System & Sex Hormone***		
Gynaecological Anti infectives And Antiseptics	8	0.23
Other Gynaecologicals	6	0.17
Sex Hormones And Modulators Of Genital System	45	1.30
Urologicals	26	0.75
**Systemic Hormonal Preparation Excluding Sex**		
Pituitary And Hypothalamic Hormones and Analogues	1	0.03
Corticosteroids For Systemic Use	20	0.58
**Anti-Infectives For Systemic Use**		
Anti bacterial For Systemic Use	19	0.55
Anti mycotics For Systemic Use	22	0.64
**Anti-Neoplastic & Immunomodulating Agents**		
Immune Sera And Immunoglobulins	2	0.06
Endocrine Therapy	1	0.03
***Musculo-Skeletal System***		
Anti-inflammatory And Anti-rheumatic Products	357	10.31
Topical Products For Joints And Muscle Pains	122	3.52
Muscle Relaxants	21	0.61
Anti gout Preparations	16	0.46
Drug For Treatment Of Bone Diseases	2	0.06
Other Drugs For Disorder Of The Musculoskeletal Systems	29	0.84
***Nervous System***		
Anaesthetic	2	0.06
Analgesics	344	9.94
Psycholeptics	2	0.06
Psycho analeptics	6	0.17
Other Nervous System Drugs	16	0.46
***Anti-Parasitic Products, Insecticides And Repellents***		
Anti protozoals	5	0.14
Anti helmintics	20	0.58
Ecto parasiticides (including Scabicides, Insecticides and Repellent)	7	0.20
***Respiratory System***		
Nasal Preparations	131	3.78
Throat Preparations	7	0.20
Drugs For Obstructive Airway Diseases	39	1.13
Cough And Cold Preparations	200	5.78
Antihistamine For Systemic Use	279	8.06
Other Respiratory System Products	16	0.46
***Sensory Organs***		
Opthalmologicals	69	1.99
Orthologicals	14	0.40
***Others***		
Other Therapeutic Products	2	0.06
General Nutrients	16	0.46
All Other Non-Therapeutic Products	2	0.06

ATC: Anatomical Therapeutic Chemical.

The most frequently purchased NPM based on level one (anatomical group) ATC classification were for the alimentary tract and metabolism (31.8%), followed by respiratory system (19.4%), and musculoskeletal system (15.8%). Of alimentary tract and metabolism medicines, vitamins (281/1101 = 25.5%), drugs for acid-related disorders (156/1101 = 14.2%), and mineral supplements (140/1101 = 12.7%) were among the most frequently purchased NPM. Of respiratory system medicines, antihistamines for systemic use (279/672 = 41.5%), cough and cold preparations (200/672 = 29.8%), and nasal preparations (131/672 = 19.5%) were the most frequently purchased medicines. Customers who purchased musculoskeletal system medicines mostly purchased anti-inflammatory and anti-rheumatic products (357/547 = 65.3%), topical products for joint paint and muscle pain (122/547 = 22.3%), and muscle relaxants (21/547 = 3.8%).

Regardless of the level one ATC classification (main anatomical group), the sub-analysis at level two of the ATC classification (main therapeutic group) found that there were some differences in the most frequently purchased NPM. The most frequently purchased medicines were anti-inflammatory and anti-rheumatic products (10.3%), followed by analgesics (9.9%), vitamins (8.1%), antihistamines for systemic use (8.1%), cough and cold preparations (5.8%), drugs for acid-related disorders (4.5%), and mineral supplements (4.0%).

### Pattern of Non-prescription Medicine Purchases

Table 3 summarizes the pattern of NPM purchases based on the demographic characteristics of the purchasers. There was a significant difference between types of medicine purchased by rural and urban residents [χ2 (df = 13) = 69.35, p<0.01]. Rural customers purchased more cardiovascular system (3.9% vs. 3.1%) and musculoskeletal system medicines (20.9% vs. 14.4%) than urban customers. The latter purchased more alimentary tract and metabolism medicines (32.1% vs. 30.6%) and dermatological medicines (10.9% vs. 6.7%) than rural customers.

**Table 3:** Pattern of non-prescription medicine by customers’ demographic.

Variables	Types of Non-Prescription Medicine (ATC level 1)																											
	1		2		3		4		5		6		7		8		9		10		11		12		13		14	
	N	%	N	%	N	%	N	%	N	%	N	%	N	%	N	%	N	%	N	%	N	%	N	%	N	%	N	%
***Locality Of Pharmacy***																												
Rural	230	30.6	4	0.5	29	3.9	50	6.7	17	2.3	15	2.0	8	1.1	0	0.0	157	20.9	69	9.2	1	0.1	151	20.1	16	2.1	4	0.5
Urban	871	32.1	24	0.9	85	3.1	295	10.9	68	2.5	6	0.2	33	1.2	3	0.1	390	14.4	301	11.1	31	1.1	521	19.2	67	2.5	16	0.6
***Age Group***																												
25 Years And Below	149	28.2	2	0.4	11	2.1	85	16.1	12	2.3	2	0.4	8	1.5	0	0.0	63	11.9	71	13.4	4	0.8	101	19.1	15	2.8	6	1.1
26 To 40 Years	407	30.9	10	0.8	33	2.5	139	10.5	42	3.2	3	0.2	13	1.0	1	0.1	177	13.4	138	10.5	23	1.7	298	22.6	29	2.2	6	0.5
41 To 60 Years	438	32.8	8	0.6	59	4.4	102	7.6	26	1.9	11	0.8	16	1.2	2	0.1	251	18.8	142	10.6	5	0.4	236	17.7	35	2.6	5	0.4
More Than 60 Years	107	38.5	8	2.9	11	4.0	19	6.8	5	1.8	5	1.8	4	1.4	0	0.0	56	20.1	19	6.8	0	0.0	37	13.3	4	1.4	3	1.1
***Gender***																												
Male	534	30.7	17	1.0	46	2.6	156	9.0	34	2.0	12	0.7	23	1.3	2	0.1	294	16.9	183	10.5	18	1.0	380	21.8	33	1.9	10	0.6
Female	567	33.0	11	0.6	68	4.0	189	11.0	51	3.0	9	0.5	18	1.0	1	0.1	253	14.7	187	10.9	14	0.8	292	17.0	50	2.9	10	0.6
***Ethnicity***																												
Malay	546	28.9	12	0.6	49	2.6	199	10.5	55	2.9	16	0.8	34	1.8	2	0.1	324	17.2	230	12.2	21	1.1	354	18.8	43	2.3	3	0.2
Chinese	365	38.1	14	1.5	49	5.1	80	8.4	16	1.7	3	0.3	1	0.1	1	0.1	132	13.8	77	8.0	7	0.7	175	18.3	26	2.7	12	1.3
Indian	120	35.7	1	0.3	10	3.0	31	9.2	4	1.2	1	0.3	4	1.2	0	0.0	44	13.1	35	10.4	3	0.9	71	21.1	9	2.7	3	0.9
Others	69	24.7	1	0.4	6	2.2	35	12.5	10	3.6	1	0.4	2	0.7	0	0.0	47	16.8	28	10.0	1	0.4	72	25.8	5	1.8	2	0.7
***Education Level***																												
No Formal Education	44	32.6	1	0.7	4	3.0	10	7.4	3	2.2	0	0.0	2	1.5	0	0.0	28	20.7	12	8.9	0	0.0	28	20.7	3	2.2	0	0.0
Primary Education	165	33.7	2	0.4	14	2.9	44	9.0	8	1.6	7	1.4	5	1.0	0	0.0	115	23.5	40	8.2	4	0.8	75	15.3	9	1.8	1	0.2
Secondary Education	566	31.0	16	0.9	62	3.4	171	9.4	50	2.7	13	0.7	21	1.2	1	0.1	293	16.1	193	10.6	18	1.0	361	19.8	46	2.5	12	0.7
Tertiary Education	326	32.1	9	0.9	34	3.3	120	11.8	24	2.4	1	0.1	13	1.3	2	0.2	111	10.9	125	12.3	10	1.0	208	20.5	25	2.5	7	0.7
***Occupation***																												
Senior Officials And Manager	73	36.0	3	1.5	11	5.4	20	9.9	5	2.5	0	0.0	0	0.0	1	0.5	33	16.3	24	11.8	3	1.5	26	12.8	3	1.5	1	0.5
Professionals	160	31.2	5	1.0	16	3.1	65	12.7	18	3.5	1	0.2	1	0.2	1	0.2	57	11.1	61	11.9	4	0.8	110	21.4	13	2.5	1	0.2
Technicians And Associate Professionals	65	31.3	2	1.0	4	1.9	16	7.7	6	2.9	0	0.0	7	3.4	0	0.0	20	9.6	29	13.9	1	0.5	53	25.5	5	2.4	0	0.0
Clerical Works	72	28.3	2	0.8	5	2.0	28	11.0	4	1.6	2	0.8	2	0.8	0	0.0	25	9.8	40	15.7	5	2.0	63	24.8	6	2.4	0	0.0
Service Workers, Shop And Market Sales Workers	160	30.5	3	0.6	14	2.7	48	9.2	11	2.1	2	0.4	6	1.1	0	0.0	91	17.4	56	10.7	4	0.8	114	21.8	12	2.3	3	0.6
Craft And Related Trades Worker	37	30.3	1	0.8	3	2.5	10	8.2	2	1.6	6	4.9	1	0.8	0	0.0	27	22.1	8	6.6	0	0.0	24	19.7	2	1.6	1	0.8
Skilled Agricultural And Fishery Workers	53	34.9	2	1.3	3	2.0	13	8.6	3	2.0	1	0.7	2	1.3	0	0.0	25	16.4	12	7.9	2	1.3	32	21.1	3	2.0	1	0.7
Plant And Machine-Operator And Assembler	25	25.3	1	1.0	2	2.0	9	9.1	2	2.0	0	0.0	2	2.0	0	0.0	22	22.2	10	10.1	0	0.0	20	20.2	6	6.1	0	0.0
Elementary Occupation	94	32.4	0	0.0	4	1.4	27	9.3	8	2.8	1	0.3	4	1.4	0	0.0	79	27.2	21	7.2	4	1.4	41	14.1	7	2.4	0	0.0
Students	72	29.1	0	0.0	6	2.4	39	15.8	6	2.4	0	0.0	5	2.0	0	0.0	25	10.1	32	13.0	2	0.8	52	21.1	4	1.6	4	1.6
Retiree	70	37.2	6	3.2	12	6.4	12	6.4	3	1.6	3	1.6	3	1.6	0	0.0	32	17.0	16	8.5	0	0.0	28	14.9	2	1.1	1	0.5
Not Working/Housewife	201	33.0	3	0.5	33	5.4	55	9.0	16	2.6	3	0.5	8	1.3	1	0.2	97	15.9	56	9.2	7	1.1	106	17.4	16	2.6	8	1.3
***Household Income Per Month***																												
below RM500	66	32.4	3	1.5	4	2.0	25	12.3	8	3.9	1	0.5	6	2.9	0	0.0	40	19.6	18	8.8	0	0.0	29	14.2	4	2.0	0	0.0
RM500 to RM1000	148	26.4	0	0.0	8	1.4	65	11.6	16	2.9	6	1.1	8	1.4	0	0.0	118	21.0	66	11.8	7	1.2	106	18.9	11	2.0	2	0.4
RM1001 to RM2000	246	28.5	8	0.9	23	2.7	66	7.6	21	2.4	7	0.8	9	1.0	0	0.0	152	17.6	104	12.1	5	0.6	196	22.7	21	2.4	5	0.6
RM2001 to RM3000	234	31.8	3	0.4	24	3.3	84	11.4	14	1.9	4	0.5	12	1.6	1	0.1	103	14.0	72	9.8	13	1.8	156	21.2	15	2.0	2	0.3
RM3001 to RM4000	152	33.8	7	1.6	23	5.1	44	9.8	10	2.2	2	0.4	4	0.9	0	0.0	51	11.3	54	12.0	5	1.1	85	18.9	10	2.2	3	0.7
RM4001 to 5000	74	35.1	6	2.8	12	5.7	17	8.1	8	3.8	0	0.0	0	0.0	1	0.5	34	16.1	25	11.8	0	0.0	24	11.4	10	4.7	0	0.0
More than RM5000	172	41.6	1	0.2	17	4.1	44	10.7	6	1.5	1	0.2	2	0.5	1	0.2	46	11.1	28	6.8	2	0.5	73	17.7	12	2.9	8	1.9

1 = Alimentary Tract & Metabolism, 2 = Blood & Blood Forming Organs, 3 = Cardio-Vascular System, 4 = Dermatological, 5 = Genito-Urinary System & Sex Hormone, 6 = Systemic Hormonal Preparation Excluding Sex, 7 = Anti-Infectives For Systemic Use, 8 = Anti Neoplastic & Immune Modulating Agents, 9 = Musculo-Skeletal System, 10 = Nervous System, 11 = Anti Parasitic, Insecticides And Repellent, 12 = Respiratory System, 13 = Sensory Organs, 14 = Others.

There was also a significant difference in the selection of NPM among different age groups [χ2 (df = 39) = 149.17, p<0.01]. Elderly people (more than 60 years old) purchased mostly alimentary tract and metabolism medicines, musculoskeletal system medicines, and blood and blood-forming organ medicines. Younger customers (25 years and below) mostly purchased dermatological medicines (149, 28.2%), anti-infectives for systemic use (101, 19.1), and nervous system medicines (85, 16.1).

The selection of NPM also differed significantly between men and women [χ2 (df = 13) = 32.93, p = 0.002]. Men mostly purchased musculoskeletal system medicines, respiratory system medicines, and anti-infectives for systemic use. Women purchased more alimentary tract and metabolism medicines, cardiovascular system medicines, and dermatological medicines.

Ethnicity was also significantly associated with type of NPM purchased [χ2 (df = 39) = 118.89, p<0.01]. Customers of Malay ethnicity mostly purchased anti-infectives for systemic use and musculoskeletal system medicines, while Chinese customers purchased more alimentary and cardiovascular system medicines. Indian customers purchased more respiratory system medicines than other groups.

Level of education was found to have a significant association with the type of NPM purchased [χ2 (df = 39) = 74.74, p<0.01]. Customers with a tertiary level of education purchased more dermatological and nervous system medicines compared to other groups. Customers who had not received any formal education purchased more musculoskeletal system medicines, anti-infectives for systemic use and respiratory system medicines.

Type of occupation was also significantly associated with the selection of NPM [χ2 (df = 143) = 280.70, p<0.01]. Musculoskeletal system medicines were commonly purchased by those subjects who working in a field of elementary occupations, dermatological medicines by students, and alimentary tract and metabolism medicines by senior officials, managers, and retirees.

Household income per month was also associated with the type of NPM purchased [χ2 (df = 78) = 190.26, p<0.01]. Customers with a household income less than RM500 per month were mostly purchased dermatological and anti-infectives for systemic use. High earners (more than RM5000 per month) mostly purchased medicines in the alimentary tract and metabolism group.

### Predictors of Non-prescription Medicines Purchased

Table four describes the results of multinomial logistic regression analysis. The regression was performed to identify the predictors of NPM purchased. Locality (urban or rural), gender, age, ethnicity, type of occupation and household income per month were the demographic factors that significantly predicted the purchase of NPM. The pseudo R-square analysis (Cox and Snell) found that 20% of the variation in NPM purchased is explainable by the demographic variables of the respondents.

**Table 4:** Results of multinomial logistic regression analysis to determine predictors for type of non-prescription medicines purchased.

	Parameters			
	−2 Log Likelihood	Chi-Square	df	P value
***Model Fitting Information***				
Intercept Only	11642.851			
Final	10897.313	745.539	377	<0.001
***Goodness-of-Fit***				
Pearson		25046.189	27976	1.000
Deviance		9785.437	27976	1.000
***Likelihood Ratio Tests***				
Intercept	10897.313	0.000	0	
Number of family member	10905.691	8.378	13	0.818
Locality	10953.044	55.731	13	<0.001
Gender	10929.871	32.558	13	0.002
Ethnicity	10981.573	84.261	39	<0.001
Types of occupation	11071.043	173.730	143	0.041
Household income per month	11047.418	150.106	78	<0.001
Age	11583.265	75.952	13	<0.001
Education level	10934.000	36.687	39	0.576

* Pseudo R square (Cox and Snell = 0.198).

## Discussion

This study was conducted to investigate and explore the pattern of NPM use among Malaysian citizens who utilized the community pharmacies. Similar studies have been conducted in developed countries such as New Zealand and Australia. The findings of such studies are important in pharmaceutical marketing and to explore the practice of self-medication with NPM [5].

In the current study, the NPM purchased were categorized according to the ATC classification, up to the second level. To our knowledge, there is currently no standardized classification of NPM. For this reason, comparison with the current NPM market and other studies is difficult. The ATC classification introduced by the World Health Organization is the ‘gold standard’ for international drug utilization research [14]. A drawback of our study was that we only used the ATC classification and not the defined daily dose (DDD). This was because the dose of the NPM that been purchased was not recorded. However, this does not affect the usefulness of the data that was gathered.

The alimentary tract and metabolism group of medicines were the most frequently purchased NPM in this study, particularly vitamins, minerals, and drugs for acid disorders. This was expected as most of the medicines in the group were either pharmacist only medicines or over-the-counter medicines. Vitamins and minerals are usually purchased as dietary supplements. The increasing use of these products in Malaysia can be explained in part by the heightened awareness on health and general well being in recent years. The direct to consumer’s advertisement also plays a role in this situation. Furthermore, the liberalisation of Medicines (Advertisement and Sale) Act 1956 has affected the purchasing trend of NPM [15]. The Advertising Standards Authority of Malaysia functions to provide guidance via the Malaysian Code of Advertising Practice to promote and enforce high ethical standards in advertising. The Medicine Advertisement Board of the Ministry of Health regulates the advertisement of pharmaceutical products, including vitamins and dietary supplements, under the Medicines (Advertisement & Sale) Act of 1956 (revised in 1983). The Medicine Advertisements Board Regulation of 1976 was implemented to ensure responsible advertising of medicines, appliances, and remedies that can be purchased by the public without a prescription [16]. Thus; there is a need to not only monitor the purchasing trend of NPM but also the consequences of changes in direct-to-consumer advertising.

The high percentage of customers who purchased musculoskeletal system medicines such as anti-inflammatory and anti-rheumatic products and topical products for joint paint and muscle pain is alarming; particularly as anti-inflammatories were the most frequently purchased medicine and most anti-inflammatories consist of non-steroidal anti-inflammatory drugs. The misuse of such medicines could lead to potential risks such as gastrointestinal bleeding and renal impairment [17], [18]. Thus, the selling of NPM must be monitored periodically to ensure rational utilization of these medicines.

Antihistamines for systemic use, cough and cold preparations, and nasal preparations were the most frequently purchased medicine in the respiratory system group. As the utilization of NPM reflects self-medication practices, there is a risk of potential misuse of medicines and a potential delay in treating serious medical conditions. A study on the intention and frequency of use of medicines should be carried out to determine the magnitude of the misuse of NPM in our society.

This study also identified the association between occupation and NPM selection besides the demographic factors that had been evaluated in many other studies. The elementary occupations in the list include cleaners and helpers, agricultural, forestry and fishery labourers, and labourers in the mining, construction, manufacturing, and transport sectors. These groups are at high risk of developing musculoskeletal disorders such as repetitive strain injury (RSI), occupational overuse syndrome (OOS), and cumulative trauma disorder (CTD) [19]. This may explain the frequent purchase of musculoskeletal system medicines among those working in elementary occupations. Many studies have reported the high risk of myocardial infarction among senior officials and managers [20]. The frequent purchase of alimentary tract and metabolism medicines such as drugs for acid-related disorders is worrying. Confusion between cardiovascular disease symptoms and gastrointestinal disorders might be an underlying cause. Appropriate consultation and symptom assessment by community pharmacists would help customers to select the correct non-prescription medicine and thus avoid this problem.

This study found that the predictors of NPM purchase in Malaysian customers were locality (urban or rural), gender, age, ethnicity, type of occupation and household income per month. These factors predicted about 20% of the NPM purchased. A factor that was not studied was the health status of the customers. Another important aspect that should be studied further was for whom the medicine was intended and for what indication. Our recommendation is to incorporate such factors in further research in the near future.

### Conclusion

Malaysian consumers mainly purchased NPM to treat problems with the alimentary tract and metabolism. The choice of NPM was associated with socio-demographic profile. The predictors of NPM purchase were locality (urban or rural), gender, age, ethnicity, type of occupation and household income per month. The development of a national policy on NPM should be supported to ensure good self-medication practice among the Malaysian population. Future studies need to explore the safety and effectiveness of using these NPM.

The study focused on NPM purchased and did not inquire the intent of NPM purchased. Furthermore, paucity of information towards NPM purchase restricted the analysis to second level of ATC. Another limitation of the study is that it did not report defined daily dose.
